# Segmentation of sputum smear images for detection of tuberculosis bacilli

**DOI:** 10.1186/1471-2334-12-S1-O14

**Published:** 2012-05-04

**Authors:** Feminna Sheeba, Robinson Thamburaj, Joy Sarojini Michael, P Maqlin, Joy John Mammen

**Affiliations:** 1 Madras Christian College, Chennai, India; 2 Christian Medical College, Vellore, India

## Background

Tuberculosis (TB) is a common and lethal infectious disease, which requires accurate and early diagnosis for effective containment. Essential for the diagnosis of pulmonary infection is the detection of the bacilli through the manual microscopic examination of ZN-stained sputum smear, which is a time-consuming, complex process necessitating at least 8-10 minutes per slide. Moreover, the quality of the detection is highly subjective to the individual who performs the analysis. These results can clearly be improved upon by using image processing techniques. The proposed work uses the segmentation techniques to automate the analysis of the sputum smear images and to detect the presence of tuberculosis bacilli in them.

## Methods

This study involves assessing ZN-stained images obtained using a digital camera DFC280 attached to a compound microscope. The images acquired are 24-bit coloured tiff images. The software was developed using MATLAB R2009b. It has been deduced from the images, that the bacilli have a distinct colour and shape. The aim of the study is to identify the rod-shaped coloured bacilli, which are 1-10μm long. Colour based segmentation and property information, combined with morphological operations are used for bacilli detection.

## Results

A prototype application was developed to identify these bacilli. The sensitivity, specificity, PPV and NPV of the study is 68.75%, 93.75%, 91.67% and 75% respectively.

## Conclusions

The above study shows the potential in harnessing image analysis techniques for the detection and study of the TB bacilli. Texture analysis and identifying overlapping bacilli are required for more accurate results.

